# Prediction of protein long-range contacts using an ensemble of genetic algorithm classifiers with sequence profile centers

**DOI:** 10.1186/1472-6807-10-S1-S2

**Published:** 2010-05-17

**Authors:** Peng Chen, Jinyan Li

**Affiliations:** 1 Bioinformatics Research Center, School of Computer Engineering, Nanyang Technological University, Singapore 639798

## Abstract

**Background:**

Prediction of long-range inter-residue contacts is an important topic in bioinformatics research. It is helpful for determining protein structures, understanding protein foldings, and therefore advancing the annotation of protein functions.

**Results:**

In this paper, we propose a novel ensemble of genetic algorithm classifiers (GaCs) to address the long-range contact prediction problem. Our method is based on the key idea called sequence profile centers (SPCs). Each SPC is the average sequence profiles of residue pairs belonging to the same contact class or non-contact class. GaCs train on multiple but different pairs of long-range contact data (positive data) and long-range non-contact data (negative data). The negative data sets, having roughly the same sizes as the positive ones, are constructed by random sampling over the original imbalanced negative data. As a result, about 21.5% long-range contacts are correctly predicted. We also found that the ensemble of GaCs indeed makes an accuracy improvement by around 5.6% over the single GaC.

**Conclusions:**

Classifiers with the use of sequence profile centers may advance the long-range contact prediction. In line with this approach, key structural features in proteins would be determined with high efficiency and accuracy.

## Background

Proteins have complicated three-dimensional (3D) structures. It is often cost-expensive and speed-slow for proteins to be resolved by experimental techniques, such as x-ray crystallography and nuclear magnetic resonance (NMR). This is why more than ten million proteins are sequenced, while only 62,000 protein structures are stored in PDB. As protein structures are the basis for understanding protein functions and rational molecules design, associative studies between protein sequences and 3D structures by computational techniques have received increasing research interests.

It is known that non-local interactions of residue pairs are crucial for proteins to attain their native state [1,2]. Fariselli and Casadio reported that if residue contacts for a protein are known, the major features of its 3D structure could be determined by combining the correctly predicted motifs of secondary structures [3]. To a more extend, even a corrupted map with nonphysical contacts of a protein could lead to the recovery of its 3D structure by projecting the contact map onto its closest physical structural counterpart [4]. Previous results also indicate that 50% correctly predicted contacts ought to suffice that reconstruction [5] at least for proteins with less than 150 amino acids and with 8Å distance cutoff.

There have emerged various methods addressing the inter-residue contact prediction problem, such as methods with the use of evolutionary information [6], a Self-Organizing Map (SOM) integrated by genetic programming (GP) [7], neural networks (NN) [8,9], general input-output hidden Markov models (GIOHMMs) [5], support vector machines (SVMs) [10,11] and so on. Punta and Rost reported that about 30% of the predicted contacts were correct (in accuracy) with the residue separation at least six residues, where about 10% of the observed contacts are predicted (in coverage) [12]. Vullo's two-stage predictor achieved 19.8% prediction accuracy for the minimum contact separation of 24 residues, when evaluating on the top* L*/5 predicted contacts [13]. Wu and Zhang conducted a comprehensive assessment on sequence-based and template-based methods for contact map prediction and achieved an accuracy around 20% for long-range contacts [14]. Currently, the most accurate contact predictor, named NNcon, achieved 18% accuracy based on the CASP8 dataset [15]. In spite of great progress in the prediction of inter-residue contacts, the development of computational approaches is still at its embryonic stage. Therefore, fully exploring inter-residue contacts in proteins and designing novel approaches is highly demanded.

In this paper, we propose an ensemble of genetic algorithm classifiers (GaCs) to study the problem of long-range contact prediction. The input to this ensemble classifier are sequence profile centers (SPCs). Each SPC represents the average sequence profiles of residue pairs that belong to the same long-range contact class or long-range non-contact class [16]. One sequence profile is an encoding vector for a residue pair whose spatial distance between the two members falls into one distance interval ≤ 8Å or > 8Å, and whose residue separation is ≥ 24 in the sequence. Our GaCs train on multiple but different pairs of long-range contact data (positive data) and long-range non-contact data (negative data). The negative data sets, having roughly the same sizes as the positive ones, are constructed by random sampling over the original imbalanced negative data. As a result, about 21.5% long-range contacts are correctly predicted. We also found that the ensemble of GaCs indeed makes an accuracy improvement by around 5.6% over the single GaC.

## Results and discussion

**Figure 1:**
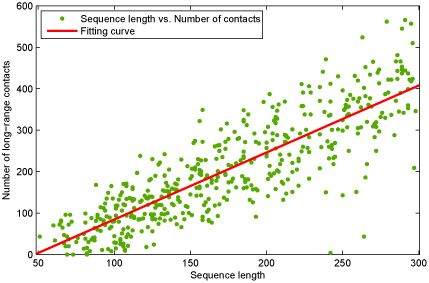
**Relationship of the number of long-range contacts versus the corresponding protein sequence length**. The red line denotes the fitting curve of sequence length against the number of long-range contacts for protein chains.

Our dataset involves 480 protein chains with 83307 residues, and it consists of 97639 residue pairs in long-range contact. Some proteins have more residue pairs in long-range contact and some ones contain fewer such residue pairs with respect to protein sequence length. However, protein sequence length has an approximately linear relationship with the number of long-range contacts [17]. Figure 1 shows such relationship (the red line in Figure 1).

**Figure 2:**
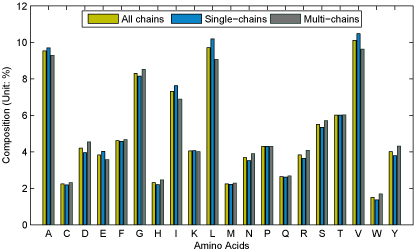
**Amino acid compositions for multi-chain proteins and those with single-chain**. The sky-blue bar stands for protein with single chain and the grey one denotes multi-chain protein. In addition, all protein chains are colored in brown.

Gromiha* et al* found that residue Ile is the most possible residue occurring in long-range contacts followed by Cys, Val, Tyr, Trp, Phe and Leu [2], indicating that hydrophobic residues mainly influence the long-range contacts. This also holds roughly true for our dataset. Figure 2 illustrates the amino acid composition for long-range contacts based on our dataset. Residues Val, Leu, Ala, Gly and Ile are more likely to be in long-range contact than other residue types. Conversely, residue Trp has the least long-range contact possibility followed by Cys, His, Met and Gln. In Figure 2, amino acids have almost the same long-range contact preference for multi-chain proteins as those with single-chain, which suggests that the statistics of amino acid composition is very consistent.

**Figure 3:**
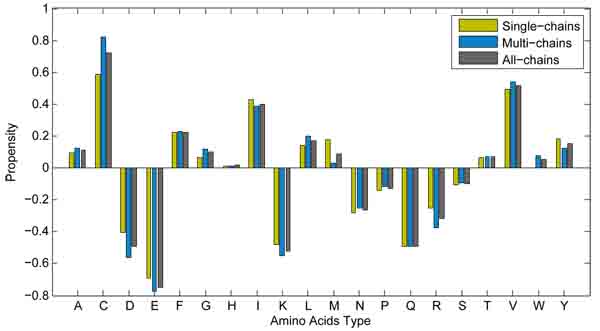
**Propensities of amino acid types in long-range contacts to the types in long-range non-contacts for multi-chain proteins and those with single-chain.** The sky-blue bar stands for protein with single chain and the grey one denotes multi-chain protein. In addition, brown colored bar is for averaging all protein chains.

We also investigate the propensities of amino acid types in long-range contacts to the types in long-range non-contacts. Each amino acid type in long-range contacts or in long-range non-contacts is respectively counted. The composition of amino acid type in long-range contacts or in long-range non-contacts is the ratio of the amount of individual amino acid type to that of total amino acids in long-range contacts or in long-range non-contacts. The propensity difference of each amino acid type is the ratio of the percentage of the type in contacts to that in non-contacts. These propensities for all amino acid types are shown in a logarithm (*log_2_*) scale in Figure 3. We can note that the larger the values of amino acid types are, the more possible they are in long-range contacts, while those with smaller values are more possibly in long-range non-contacts. Amino acids with larger propensity values, such as 'C', 'V', 'I', and 'L', representing hydrophobicity, are always in long-range contacts. However hydrophilic amino acids 'E', 'D', and 'K' often appear in long-range non-contacts. More importantly, cysteine and valine are the most frequently occurring residue in long-range contacts, but the glutamic acid appears in long-range non-contacts mostly.

### Transformation of sample vectors

**Figure 4:**
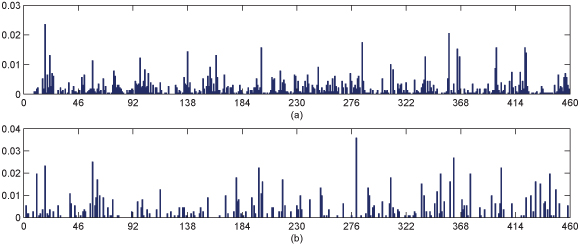
**A transformation case of input vector by one classifier.** (a) A case of original input vector with 460 dimensions, (b) Transformed input vector by the transformation for contact class 1 classifier. All the two vectors are equalized by normalized themselves.

GA was applied to reduce the dimensionality of input vector, the reduced (transformed) input vectors were then used as the input vectors of our classifier. In the transformation, some input variables were removed or merged, but it was done without decreasing the information of input. One computational benefit of this transformation is that the computational cost was dropped dramatically. For the two-class problem in our experiments, two optimal non-linear transformations for the two corresponding sub-classifiers were achieved. Therefore, there are two-discards for representing the ratio of the number of removed variables to the total one. For instance, one discard ratio 34.348% for a sub-classifier is illustrated in Figure 4. In this case, there are 143 original variables removed, and 65 variables merged together into only 40 variables. A transformed vector can be obtained by normalizing itself after removing or merging the original variables. Other transformed vectors can be got in the similar way illustrated in Figure 4. 

### Performance of the GaCs ensemble

One GaC predictor can predict whether or not one sample vector belongs to long-range contact class. In this work there are 20 individual GaCs based on pairs of positive training sample set and different negative training sample subsets without overlap (refer to Methods section for details, where *N* = 20 in this work). A set of parameters is used to implement each individual GaC. As discussed in the method section, each protein in the dataset is represented as a vector of features. Feature vectors in the training dataset are first encoded into a string of chromosome. Each individual (or chromosome string) of each genetic classifier is a vector of the same size as the feature vector (460 dimensions in this work). 150 individuals compose the initial population of our genetic algorithms. Individuals of top 5% fitness values are selected to the next generation directly, while the others will go through the crossover and mutation procedures based on the preset crossover (0.95%) and mutation (0.01%) probabilities. After a number of iterations, each genetic algorithm terminates when the best fitness score did not change over 120 iterations and the best individuals are then obtained. The best individual is considered to be the best feature transformer for the prediction of long-range contacts. Other parameters associated with the implementation of genetic algorithm classifier are listed here: the crossover type is single point and the roulette wheel technique is used as the selector type.

To show the performance of the combination of several GaCs, four levels of ensembles of GaCs were constructed by the number of GaCs. Ensemble 5 denotes that there are five GaCs to be combined, while ensembles 10, 15, and 20 stand for 10, 15, and 20 GaCs to be combined to evaluate the improvement of classifiers ensemble, respectively. Table 1 shows the comparison of the four levels of GaCs ensembles. Performance of the best individual GaC is also shown in Table 1. It can be found in Table 1 that ensemble 20 outperforms other ensembles and achieves an increase of accuracy by 2%, while the best individual GaC performs worse than the four ensembles.

**Table 1:** Comparison of four levels of GaCs ensembles

Level	Residue-Residue Separation ≥ 24			
	
	2*L*	*L*	*L*/2	*L*/5
1*	3.63	4.72	7.49	15.89
5	4.93	6.23	9.85	16.68
10	6.18	6.85	9.19	18.82
15	4	6.95	11.24	19.76
20	4.87	6.94	11.06	21.49

* The best individual GaC predictor among twenty GaCs.

Since the contact prediction accuracy varies significantly with individual proteins and their structure classes [7], we calculate accuracy for each test protein based on GaCs ensemble 20. For each protein chain, we select four levels of the number of predicted contacts in the order of predicted distance between SP vector and SP center of long-range contact. The reason in doing so is that the total number of true contacts has approximately a linear relationship with the protein length [17]. The relationship was also shown in Figure 1 for our dataset. In detail, the four levels are '2L', 'L', 'L/2', and 'L/5', respectively, where L denotes protein sequence length. Results show that in many cases (e.g. 1hh7A, 1bxaA, 1gpr_, 1cewI, 1cznA, 1gn0A, 1igd_, 1tif_, 1s8nA), the prediction accuracies are larger than 30%. However, the prediction accuracies for some protein chains such as 1cv8_ and 1c7kA are pretty low. We found that the contact prediction accuracy heavily depends on the calculation of SP centers, on the number of long-range contacts and on the quality of multiple sequence alignment as well as the proportion of beta-sheets. Furthermore, in order to understand the distribution of our GaC long-range contact prediction with respect to CATH [18] domain classes, we compute the average accuracies on the five CATH structure classes (Table 2). According to Table 2, the contact prediction accuracies on proteins belonging to *β*-sheets (*α — β,* all *β*) is higher than that of all *α*-helical proteins, which is consistent with other previous observations [7,10]. In Table 2, the average accuracy is about 21.5% when evaluating the top* L*/5 predicted contacts and the residue pair with 24 apart. Taking into account the inherent physical restraints of protein structures, this prediction performance may be helpful for reconstructing an* ab initio* low-resolution structure since previous experiments showed that only* L*/5 or even less residues contacts are required to reconstruct a low resolution structure for a small protein [19-23]. However, the hard challenge is how to reconstruct a protein structure from even a corrupted predicted contact map [4], where contact restraints are much less reliable than the experimental contacts determined by NMR techniques.

**Table 2:** Performance comparison on CATH classes

CATH Class	Sequence Length	Classification Accuracy (%)				ratio*	Protein Number
				
		2*L*	*L*	*L*/2	*L*/5		
Alpha	<100	6.1	10.69	18.6	30.67	2.53	14
	100-200	4.57	7.41	10.44	23.43	1.53	30
	>200	5.34	7.32	11.06	16.74	0.96	27
	
	Average	5.16	8.02	12.28	22.31	1.51	71

Beta	<100	8.9	10.66	17.18	35.44	6.03	14
	100-200	5.72	8.33	13.5	30.81	2.68	56
	>200	5.03	7.62	13.2	28.23	1.95	35
	
	Average	5.92	8.41	13.89	30.57	2.88	105

Alpha Beta	<100	7.39	7.81	13.98	25.98	5.56	30
	100-200	4.77	6.93	10.63	24.11	2.23	99
	>200	3.8	5.54	8.75	15.69	1.39	112
	
	Average	4.65	6.39	10.17	20.43	2.25	241

Few SS**	<100	9.03	13.89	14.71	31.71	2.02	1
	100-200	5.43	9.15	14	14.29	1.2	3
	>200	4.92	7.92	10.67	13.79	0.68	2
	
	Average	5.86	9.53	13.01	17.03	1.16	6

Multi-domain chains	<100	4.71	7.69	10.35	12.5	2.3	3
	100-200	3.42	4.88	8.25	9.34	1.58	28
	>200	3.21	4.77	7.18	7.7	1.14	26
	
	Average	3.39	4.98	7.88	8.76	1.42	57

All***	<100	7.34	9.2	15.58	28.61	4.77	62
	100-200	4.82	7.12	11.09	23.7	2.15	216
	>200	4.16	6.06	9.65	16.96	1.39	202
	
	**Average**	**4.87**	**6.94**	**11.06**	**21.49**	**2.17**	**480**

*The ratio of the number of residue pairs in long-range contact to that of total long-range residue pairs.

**Protein chains containing few secondary structures.

***All protein chains in our dataset.

### Performance with respect to the number of long-range contacts in proteins

Protein chains often have different numbers of long-range contacts. However, it can be seen from Table 2 that there are no evidences to conclude that the performance on proteins containing more long-range contacts can be better than that containing less long-range contacts. On the other hand, protein chains with too few long-range contacts may worsen the classification performance. Interestingly, our model on protein chains with shorter sequence length performs better probably because proteins with shorter sequence contain larger ratio of the number of residue pairs in long-range contact to the total long-range residue pairs. For instance, the classification performance increases slightly from long sequence proteins to short proteins when considering the top L/5 classified contacts. The detailed accuracies are from 28.23% (the corresponding protein sequences are from 200 to 300), 30.81% (the corresponding protein sequences are from 100 to 200) to 35.44% (the corresponding protein sequences are less than 100) for proteins in the beta class in CATH.

Moreover, it can be seen that the larger long-range contact ratios the tested proteins have, the better predictions of inter-residue long-range contact our model probably makes. In particular, our model evaluated on the proteins in the beta and alpha-beta classes, which consist of larger long-range ratios than those in other classes, outperforms that on other proteins.

### Performance comparison based on CASP7 evaluation

The CASP7 evaluation procedure is focused on inter-residue contact predictions with linear sequence separation ≥ 12 and ≥ 24, respectively [24,25], while in this work we only focus on long-range contact prediction with linear sequence separation ≥ 24 and with assessing the top *L*/5 predicted contacts, where *L* is protein sequence length. These evaluation metrics are also similar to those used in the previous Critical Assessment of Fully Automated Structure Prediction Methods (CAFASP) [26,27] and in the EVA contact evaluation server [28]. We use the similar procedure and the same test proteins to evaluate the accuracy and coverage for our GaCs ensemble.

Contact map predictors participating in CASP7 include BETApro [29], Distill [30], GPCPRED [7], PROFcon [12], Possum [31], SAM_T06_server [32], SVMcon [10] and so on. Table 3 reports the performance of the seven automated contact map predictors in the CASP7 experiment. The performance of our GaCs ensemble is appended at the right end of Table 3. It can be seen that its accuracy is 20.7%, overall just slightly behind Possum. Its coverage at a sequence separation threshold of 24 is 3.1%, which is less than SAM-T06 and BETApro.

**Table 3:** Performance comparison based on CASP7 evaluation

	BETApro	Distill	GPCPRED	Possum	PROFcon	SAM_T06_server	SVMcon	GaCs
Acc	19.7	13.7	10.5	21.4	8.1	18.5	13.1	20.7
Cov	3.2	1.4	2	2.6	1.6	3.9	2.8	3.1

Note that some data are extracted from Table 3 in literature [10]. The eight predictors are evaluated on the 13 CASP7 domains which can be downloaded at the CASP7 web site (http://predictioncenter.org/casp7/Casp7.html). The evaluation was based on the top* L*/5 predicted contacts whose corresponding residue pair separating no less than 24 residues in sequence, where* L* is the length of the protein chain.

As previously discussed [10], one thing should be noted that in the CASP7 experiment, methods being made predictions for part of domains, such as PROFcon, can not be directly compared with other methods. Here we include its results for completeness in Table 3. Additionally, since the evaluation dataset and scheme we used may be slightly different from the official CASP7 evaluation, our purpose is to try to evaluate the current state of the art of long-range contact predictors instead of ranking them. Previous works also indicate that prediction accuracy of 50% for distant contacts with 8Å distance cutoff ought to suffice to reconstruct 3D protein structure, at least for proteins with less than 150 amino acids [5,24]. Other results showed that the accuracy level of about 30% is required for deriving moderately accurate (low resolution) 3D protein structures from scratch [19-23]. Despite the lower accuracy and coverage made by protein contact predictors, it is an important step towards reaching the accurate level [5,12,24]. From previous CASP prediction results, it can found that in a word, these predictors tend to perform more and more better [10].

Actually, in this work, the contact prediction accuracy is related to the SP centers, the number of long-range contacts and the quality of multiple sequence alignment as well as the proportion of beta-sheets. However, it is extremely difficult to build a specific non-linear expression based on the relationship. 

### Prediction results with respect to sequence profile centers

**Figure 5:**
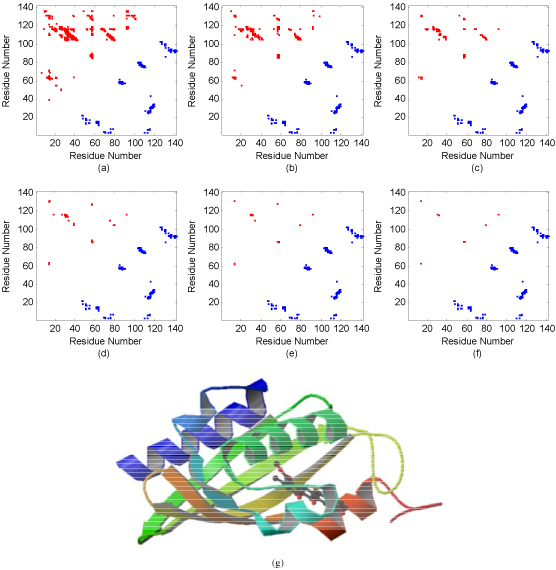
**Contact map and predicted long-range contacts for protein chain PDB:1sjwA.** (a) The comparison of contact map and predicted contacts when the top* 2L* predictions are selected; (b), (c), (d), (e) and (f) illustrate the similar comparisons when the top *L*,* L*/2, *L*/5, *L*/10 and *L*/20 predictions are selected, respectively. In addition, subgraph (g) visualizes the natural 3D structure of protein chain PDB:1sjwA. The blue square denotes the actual long-range contact and the red square indicates the predicted contact.

Prediction results on SP centers suggest that many LRCs are located around their SP centers. To illustrate the prediction results of the GaCs ensemble on SP centers, chain 'A' of protein PDB:1sjw with 142 residues was taken as an example and shown in Figure 5. Protein chain 1sjwA belongs to alpha-beta class in CATH and consists of 5 helices and 5 strands, and it contains 138 long-range contacts inter-residues and 6883 long-range non-contacts. In Figure 5, only residue pairs in long-range contact were displayed and those with sequence separation less than 24 residues were discarded. From Figure 5, most long-range contacts are predicted and a high classification accuracy is obtained. Mapping the input vectors (SP vectors) of these long-range contact residues onto one high-dimensional space, most contacts are neighboring around their corresponding high-dimensional SP center. In a similar manner, SPs for long-range non-contacts are also clustering together and distantly separated from their SP center but not shown in this work. In this case, the top *L*/5 (28) predicted contacts, whose residue members are separated no less than 24 residues in sequence, are selected and the corresponding prediction accuracy is 46.43%. In the cases of selecting the top 2*L*, *L*, *L*/2, *L*/10 and *L*/20 predicted contacts, our model achieves accuracies of 21.13%, 31%, 42.25%, 57.14%, and 63.39%, respectively. Many long-range residues located in beta sheets are successfully predicted to be in contact. It is consistent with the discovery that residues in long-range contact in proteins belonging to beta class and alpha-beta class in CATH are more easily identified than those in other classes in CATH, as discussed in most of previous work. It is also shown that the predicted contacts are clustered around the true contacts (see from Figure 5(a) to Figure 5(f)). It is of interest that many false positive contacts are also near to true contacts. Therefore, even these contacts may be helpful for reconstructing protein structure.

## Conclusions

As pointed out by Baldi [33], a machine learning algorithm adopting a simple representation of a sequence space can be much more powerful and useful than using the original data containing all details. We found that most long-range contacts or long-range non-contacts are near to their SP centers after the feature transformation by our technique. In this work, we developed a GaC ensemble to reduce the dimensionality of input features based on SP centers. The purpose of the GaC method is to transform input vectors and select a number of features, while the combination of several GaCs is used in order to achieve higher contacts prediction. As expected, the ensemble of GaCs outperforms individual GaC and achieves an increase of accuracy by around 5.6%.

We have also made the following observations: (1) Most long-range contacts or long-range non-contacts are clustered around their SP centers by using our GaCs ensemble, when selecting the top *L*/5 classified contacts. Furthermore, it was found that about 21.5% long-range contacts can be correctly predicted under the same condition; (2) Clustering proteins with long-range contacts into a few number of clusters may lead to a higher classification performance. Likewise, long-range non-contacts also behave the similar manner. Therefore, for contact class or one non-contact class, integrating a set of SP centers with the information of predicted secondary structures or hydrophobicity might improve classification prediction; (3) Alternative classifiers can be proposed to predict long-range contacts based on predicted SP centers by radial basis function neural network or support vector regression for each protein chain.

## Methods

### Datasets and cross-validation

We obtained the protein chain set from PDB-REPRDB [34], which selects protein chains from PDB based on PDB Rel. 2007_11_14, and updated on 15 April 2009. We selected protein chains that are resolved by X-ray crystallography with resolution ≤2.0Å. The sequence identity between each two chains is less than 25%. As a result, we achieved 480 protein chains which have corresponding Consurf-Hssp files [35]. The dataset can be found at our website: http://mail.ustc.edu.cn/~bigeagle/BMCStructBio2010/index.htm. To validate our approach, a two-fold cross-validation strategy was employed to conduct our experiments. In this case, predictor was trained on one subset and tested on another one and vice versa.

### Feature spaces

We firstly encode input vectors for each pair of residues, (*i*,*j*), then respectively stretch the two residues from N- to C-termini along a protein chain. Meanwhile, two corresponding sliding windows with an odd size of window length are used to encode input vectors. They are respectively centered at residue* i* and* j*, where window length is set to 9 in our work. Due to the improvement of contacts prediction by the application of segment connecting the residues of* i* and* j *[10,12,17,36], we took a third central window with five consecutive residues centered at the residue site* int ((i+j)/2).*

We used the property of residue sequence profiles (SP) obtained from HSSP database [37] at very beginning, where each residue was represented by 20 elements whose values were evaluated from multiple sequence alignment and their potential structural homologs. As discussed above, the three windows contain* (9+5+9)=23* residues, where each residue corresponds to a sequence profile vector with 20 elements. In total, the input vector for one residue pair contains *20×23=460* elements, that is, one input vector consists of 460 features or variables.

### Definition of long-range contacts

Usually, the contact map of a polypeptide chain with sequence length *N* is represented by an *N*×*N* matrix, *CM.* It is defined in terms of spatial distances between C-alpha atoms of residues and a predefined cutoff distance *d*. Usually, *d* is set as 8Å. So contact map for two-class long-range contact can be defined as:

	(1)

where *d*(*i*, *j*) denotes the spatial distance between residues* i* and j.

In this case, two residues separated at least 24 residues in sequence are named as long-range residue pair. A pair of long-range residues in contact (class 1) is regarded as a positive sample while a pair of long-range residues in non-contact (class 0) is a negative sample.

### Description of sequence profile center

The sequence profile center (SPC) is the average of all the sequence profile vectors belonging to one contact class of a protein. The definition of sequence profile center* C_i_* in one protein chain* j* for contact class* i* is given as follows:

	(2)

where *S_i_* (*l*) denotes the* l – th* sequence profile whose corresponding residue pair is to belong to contact class *i*, and* m_i_* is the number of residue pairs in contact class *i*.

For testing our method, SP centers for test protein chains, due to unknown 3D structures, need to be extracted from the training protein chains. All test chains use the same SP centers. So, the definition of SP center* C_i_* of contact class* i* for test chains is given as follows:

	(3)

where* m* denotes the number of training protein chains.

Given a test residue pair, we calculate the distance between the SP of the residue pair and every SPC. Generally, label* i* of SPC* C_i_* is assigned to a SP if the SPC* C_i_* is the nearest to the SP than the other SPC. Some other representations for profile center or centroid can be found in literature [38].

### Genetic algorithm classifiers

In this paper, GaC predictor aims to transform original input vectors in such a way that the classification rate is significantly enhanced while retaining the efficiency and simplicity of the original vectors. It proceeds to search for an optimal transformation for the variables of input vectors based on genetic optimization. After obtaining the optimal transformation, classifier based on distance dissimilarity is used to classify test samples.

#### Chromosome encoding

Genetic algorithm [39] behaves in an analogous manner to Darwinian evolution by maintaining a population of solutions based on a fitness function, and strives to obtain the individuals with the maximum or minimum fitness value within the population. A string represents each candidate in the population, which is associated with a fitness value that reflects the capability to survive into the next generation during the evolution process.

To identify long-range contacts, let* V* be a feature space set* V =* (*v*_1_,*v*_2_,... ,*v_m_*), where* v_i_* is a feature variable and* m* is the dimension of feature vectors. Each residue pair within a protein is represented as a feature vector of* V.* We want to train a GA-based classifier that can correctly classify the feature vectors into classes* C*_1_ (long-range contact) and* C*_0_ (long-range non-contact). Our goal is to search for an optimal feature transformer T that maximizes the classification rate based on the corresponding selected features. To obtain the optimal* T*, GA is applied to search through the feature space* V* with a fitness function. To do that, firstly, a vector* v_i_* of the feature space* V* is represented as a chromosome string* S_i_.* A chromosome is composed of three kinds of* expressors* represented by characters* a*, *b*, and *c*, and the size of a chromosome is the same as a feature vector.

**Figure 6:**
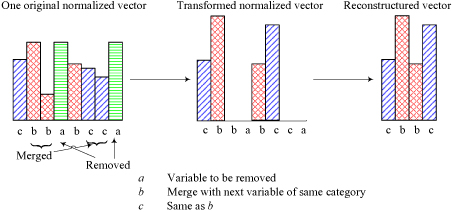
**Transformation for input vector.** One histogram bin in each original normalized vector denotes one feature or variable and the height corresponds to the magnitude of the feature. The transformed vector should be also normalized but not shown here for the clear comparison between the original vector and the resulted vector.

The schema for chromosome encoding is as follows: (1) Character* a* in a chromosome indicates that the values in the corresponding position in all feature vectors in* V* will be removed; (2) Two consecutive *b*'s or *c*'s indicate that the values in the corresponding positions will merged together. For instance, Figure 6 illustrates the transformation process for a feature vector (x_1_, x_2_, x_3_, x_4_, x_5_, x_6_, x_7_, x_8_). In this case, the corresponding chromosome is '*cbbabcca*'. After being applied the transformers, the elements of the sample feature vector being removed or merged are concatenated and normalized to form a new vector with four elements. The new normalized vector will be used for long-range contacts classification.

#### Definition of fitness function

For each transformation* T^m^* associated with the string* S_m_,* we can construct the transformed input function for input training vector *x_i_* . For class *C_k_*(*k* = 0,1), we can define the following centroid function based on* T^m^* as:

	(4)

where |*C_k_*| is the cardinality of class* C_k_*(*k* = 0, 1). *C*_1_ represents for contact class and* C*_0_ is for non-contact class.

Given these centroid functions, a new class structure  can be imposed on the input vector* x* as follows:

	(5)

where* f* is the function of the unknown model* x* and d(*,*) is a measure of dissimilarity between two functions.

Recall that the* k-th* classifier is trained to identify whether or not an input vector (unknown model) comes from class* k.* To implement the task, one fitness function for each* k-th* classifier is used to measure the discrepancies between the original class structure,* C*_1_, *C*_0_ and the imposed class structure,  based on the chromosome string* S_m_.* The fitness function is defined as:

	(6)

where  and  denote the complements of* C_k_* and , respectively. The first term counts the number of correct positive classifications, while the second term counts the number of correct negative classifications. Particularly, the maximal value of  will be obtained when the two contact class structures exactly coincide, and its value will decrease as their discrepancy increases.

#### Outputs ensemble

Statistically, the number of negative samples is much smaller than that of positive sample, which leads to a rather imbalance between positive and negative samples. To avoid the influence of imbalance problem, negative training samples are divided into several subsets without overlap by random sample selection, which have roughly the same sizes as that of positive samples. In theory, combining the outputs of a number of independent classifiers can improve classification rate since the errors made by a classifier may be corrected by the others [40-42].

A majority voting rule was adopted to combine the outputs of genetic algorithm classifiers. By using the majority voting, one input vector was predicted as positive class 1 if at least one output was labeled as positive class 1, otherwise the corresponding residue pair was in long-range non-contact class 0.

#### The flow diagram of our prediction method

**Figure 7:**
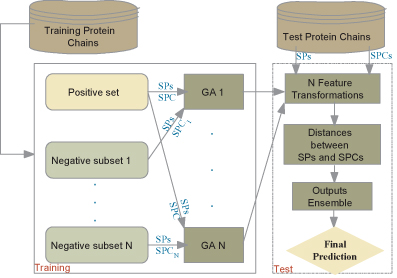
**The flow diagram of our prediction method.** SPs in the figure denote input vectors, while SPC for a training subset stand for the average corresponding SPs calculated by Equation 2 and SPCs for test protein chains are calculated by Equation 3.

Figure 7 illustrates the flowchart of our prediction method. Each GA trains on a pair of positive training dataset and a negative training subset based on corresponding SPCs (calculated by Equation 2). As a result,* N* feature transformations are achieved. Those feature transformations are applied to reduce the dimensionality of testing SPs and SPCs to be tested (calculated by Equation 3). Then, calculating the distances between transformed SPs and SPCs is to determine whether or not the corresponding residue pair is in long-range contact, and more near distance means more possible in contact inter-residue. Finally, final decision would be made by combining outputs from* N* feature transformations.

### Performance indexes

To evaluate the performance of our classifiers, we applied the criteria of accuracy (Acc) and coverage (Cov), which were adopted at CASP/CAFASP [12,43] and defined as follows:

	(7)

where* TP* denotes the number of true positives,* FP* denotes the number of false positives, and* FN* is the number of false negatives.

## List of abbreviations used

Acc: Accuracy; CATH: Class, Architecture, Topology, and Homologous superfamily; CAFASP: Critical Assessment of Fully Automated Structure Prediction; CASP: Critical Assessment of Techniques for Protein Structure Prediction; Cov: Coverage; FN: False Negative; FP: False Positive; GA: Genetic Algorithm; GaC: Genetic Algorithm Classifier; LRC: Long-Range Contact; PDB: Protein Data Bank; SP: Sequence Profile; SPC: Sequence Profile Center; TP: True Positive;

## Competing interests

The authors declare that they have no competing interests.

## Authors' contributions

Peng Chen carried out the implementation and wrote the manuscript. Jinyan Li read, revised, and approved the final manuscript.
